# A four-stage model for murine natural killer cell development in vivo

**DOI:** 10.1186/s13045-022-01243-1

**Published:** 2022-03-21

**Authors:** Shoubao Ma, Michael A. Caligiuri, Jianhua Yu

**Affiliations:** 1 Department of Hematology and Hematopoietic Cell Transplantation, City of Hope National Medical Center, Los Angeles, CA 91010 USA; 2 Hematologic Malignancies and Stem Cell Transplantation Institute, City of Hope National Medical Center, Los Angeles, CA 91010 USA; 3 Department of Immuno-Oncology, Beckman Research Institute, City of Hope, Los Angeles, CA 91010 USA; 4 Comprehensive Cancer Center, City of Hope, Los Angeles, CA 91010 USA

**Keywords:** NK cells, NK cell development, Immature NK cells, NKp46

## Abstract

Natural killer (NK) cells are the predominant innate lymphoid cells that mediate anti-viral and anti-tumor immunity. NK cells arise from hematopoietic stem cells in the bone marrow (BM) and undergo lineage specification and maturation. Despite the importance of NK cells for innate immunity and the development of innovative cancer therapy, the detailed steps linking NK progenitor (NKP) cell development through immature NK (iNK) cells to mature NK (mNK) cells are poorly defined. In this study, we found that CD49b, NK1.1, and NKp46 are sequentially acquired during the development of murine Lin^−^CD122^+^ NKP cells. Introducing NKp46 allows us to propose a four-stage developmental model, wherein CD122^+^NK1.1^−^CD49b^−^NKp46^−^ defines an NKP population, CD122^+^NK1.1^−^CD49b^+^NKp46^−^ and CD122^+^NK1.1^+^CD49b^−/+^ NKp46^−^ define iNK-a and iNK-b populations, respectively, and CD122^+^NK1.1^+^CD49b^+^NKp46^+^ defines an mNK population. These four NK cell populations are phenotypically distinct based on their expression of cell surface markers, transcription factors, and effector molecules. Using a differentiation assay ex vivo and adoptive transfer model in vivo, we confirmed that NK cell development follows our predicted four-stage model. Taken together, our findings establish two distinct populations of immature NK cells and define a model for mouse NK cell development.

**Supplementary Information:**

The online version contains supplementary material available at 10.1186/s13045-022-01243-1.

## To the Editor

Murine natural killer (NK) cells are the predominant innate lymphoid cells that arise from hematopoietic stem cells in the bone marrow (BM) and undergo lineage specification and maturation [1]. During NK cell development, a subset of multipotent hematopoietic stem cells become the common lymphoid progenitors (CLPs) [2]. CLPs give rise to pre-NK progenitor (pre-NKP) cells [3, 4], which is the earliest lineage-restricted NK cell. Pre-NKPs express the CD122 marker (IL-2 receptor β chain) and lack expression of the lineage (Lin) markers [3, 5]. In the presence of IL-15, the NKPs differentiate into immature NK (iNK) cells [5]. iNK cells further develop into mature NK (mNK) cells within the BM or egress to the periphery [6]. However, the detailed steps linking NK progenitor (NKP) cell development through immature NK (iNK) cells to mature NK (mNK) cells is poorly defined.

Over two decades ago, Rosmaraki et al. identified a three-stage NK cell developmental model where Lin^−^CD122^+^ NKPs (NK1.1^−^CD49b^−^) differentiate into iNK cells (NK1.1^+^CD49b^−^), and eventually into mature NK cells (NK1.1^+^CD49b^+^) [5]. However, the NK1.1^−^CD49b^+^ cells, which represent approximately 10% of the Lin^−^CD122^+^ population, are not included in this model and are poorly understood. Furthermore, it is not known whether additional iNK subpopulations exist. Importantly, the previous models did not incorporate NKp46, which is an exclusive NK cell marker in mice [7, 8], as the marker was not discovered at that time. Whether NKp46 is expressed in iNK cells and the sequencing of NKp46 and CD49b remains controversial [9]. To resolve the controversies about NK cell development, it is important to know the developmental stage of the NK1.1^−^CD49b^+^ cells and when NKp46 expression is initiated during NK cell development.

**Fig. 1:**
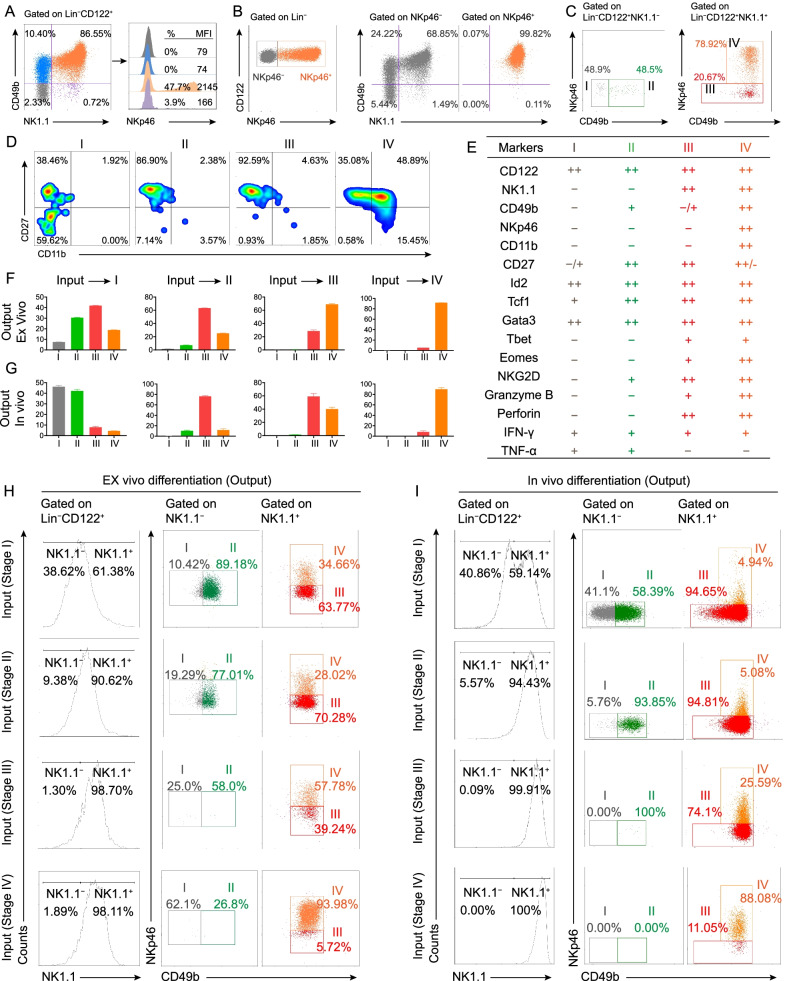
Four in vivo developmental stages of murine NK cells in the bone marrow. **A** NKp46 levels in the four populations based on NK1.1 and CD49b within Lin^−^CD122^+^ cells in the BM. **B** NK1.1 and CD49b expression in Lin^−^CD122^+^NKp46^−^ or Lin^−^CD122^+^NKp46^+^ cells in the BM. **C** Gating strategy that reveals four distinguishable subpopulations of Lin^−^CD122^+^ cells (I–IV) based on the NK1.1, CD49b and NKp46 markers. **D** CD11b and CD27 expression in populations I–IV. **E** Characteristics of four developmental stages of murine NK cells. Single-cell suspension from BM cells was prepared from wild-type mice and stained with indicated cell markers for flow cytometry. For examining IFN-γ and TNF-α, cells were stimulated with a leukocyte activation cocktail containing GolgiPlug for 4 h. The expression levels of the markers are scaled to: – (No/low expression, expression levels < 5%), + (intermediate, expression levels = 5%–50%), and +  + high, expression levels > 50%). **F**, **H** NK cell populations I–IV were sorted from NKp46^+/GFP^ reporter mice and 5 × 10^4^ cells were seeded into a 96-well plate and cultured in the presence of IL-15 (50 ng/ml) for 14 days. The cells were then harvested and analyzed using flow cytometry. Summary data (**F**) and representative dot plots (**H**) are shown for NK cell populations I–IV from NKp46^+/GFP^ reporter mice after two weeks of ex vivo differentiation (*n* = 3 per group). **G**, **I** NK cell populations I–IV from CD45.1 mice were sorted and 1 × 10^4^ to 1 × 10^5^ cells were injected intravenously into *Rag2*^−/−^*Il2rg*^−/−^ mice. The presence of transferred cells was analyzed eight weeks after adoptive transfer. Summary data (**G**) and representative dot plots (**I**) are shown for NK cell populations I–IV in the BM eight weeks after adoptive transfer (*n* = 3 per group)

We first examined the expression of NKp46 in the four populations within Lin^−^CD122^+^ cells (Fig. 1A). The NK1.1^−^CD49b^−^, NK1.1^−^CD49b^+^, and NK1.1^+^CD49b^−^ populations contained no or very few NKp46^+^ cells, whereas half of the NK1.1^+^CD49b^+^ population expressed NKp46 (Fig. 1A). Further analysis showed that the NKp46^−^ population contained NK1.1^−^ and CD49^−^ cells, whereas the NKp46^+^ population only contained NK1.1^+^CD49b^+^ cells (Fig. 1B), suggesting that NKp46 is perhaps expressed after NK1.1 and CD49b. To further clarify the developmental stages, we evaluated CD49b and NKp46 expression within NK1.1^−^ and NK1.1^+^ cells. The Lin^−^CD122^+^ population could be divided into four distinguishable populations: (I) NK1.1^−^CD49b^−^NKp46^−^, (II) NK1.1^−^CD49b^+^NKp46^−^, (III) NK1.1^+^CD49b^−/+^NKp46^−^, and (IV) NK1.1^+^CD49b^+^NKp46^+^ (Fig. 1C). The absence of NKp46^+^ cells in the NK1.1^−^ population confirms that NKp46 is expressed after NK1.1 and the presence of CD49b^+^ cells in the NK1.1^−^ population indicates that CD49b can be acquired before NK1.1. Overall, these results support a novel in *vivo* model wherein the cells in population I sequentially acquire CD49b (populations II–IV), NK1.1 (populations III and IV), and NKp46 (population IV). The presence of population III indicates overlap in the timing of CD49b and NK1.1 acquisition, though the near absence of CD49b^−^ cells in the NK1.1^+^ population supports the notion that CD49b acquisition starts first. In addition, we found that the majority of population I is CD11b^−^CD27^−^, populations II–III are mainly CD11b^−^CD27^+^, whereas approximately two-thirds of population IV are CD11b^+^ and half CD11b^+^CD27^+^ (Fig. 1D). Given that CD11b is a mature NK cell marker [6, 8], this finding is consistent with the notion that only population IV contains mature NK cells. Since population I contains both CD27^−^ and CD27^+^ subpopulations, we can further refine our model such that population I contains NKPs with CD27^−^ as an earlier stage and CD27^+^ as a later stage, but both without expression of NK1.1, CD49b, and NKp46, and CD11b, whereas populations II and III contain immature NK cells (termed iNK-a and iNK-b), which have subsequently acquired CD49b and NK1.1, respectively. Once NKp46 is acquired, they become population IV, the mNK cells, and further undergo the maturation process defined by CD11b and CD27 [6, 10], as mentioned above.

To characterize the cells in populations I–IV, we examined the effector molecules and transcription factors that control NK cell development and maturation. We found that the NKP cells expressed Id2, Tcf1 and Gata3, intermediate levels of IFN-γ and TNF-α, but did not express Tbet, Eomes, NKG2D, granzyme B, and perforin (Fig. 1E and Additional file 1: Fig. S1), consistent with their placement as NK cell progenitors in our model. The iNK-a and b cells expressed Id2, Tcf1, Gata3, low/intermediate levels of Tbet, Eomes, NKG2D, granzyme B, perforin, intermediate levels of IFN-γ, and intermediate/low levels of TNF-α (Fig. 1E and Additional file 1: Fig. S1), suggesting that they are at immature state and with limited effector functions. The mNK cells expressed almost all these functional NK cell markers, except low levels of TNF-α (Fig. 1E and Additional file 1: Fig. S1).

We next validated whether our four-stage model defines not only the markers for murine NK cell development, but also a developmental path in vivo. We sorted the four NK cell populations from NKp46^+/GFP^ reporter mice [11], and treated them with IL-15 ex vivo to stimulate NK cell development (Additional file 2)*.* As anticipated based on our model, we found that NKP cells gave rise to all three later stages of NK cells, iNK-a cells produced iNK-b and mNK cells, iNK-b cells produced mNK cells, and mNK cells maintained their phenotype and did not revert to NKP or iNK cell phenotypes (Fig. 1F, H). Next, we used adoptive transfer to place the four populations of NK cells from CD45.1 mouse into Rag2^−/−^Il2rg^−/−^ mice, which lack T, B, and NK cells but have normal levels of common gamma chain cytokines, such as IL-2, IL-7, and IL-15, that are suitable for NK cell adoptive transfer assay [12]. Transferred NKP cells gave rise to all three later populations. Transferred iNK-a cells produced iNK-b cells and mNK cells, transferred iNK-b cells only produced mNK cells, and transferred mNK cells mainly remained as mNK cells (Fig. 1G, I). Taken together, our results indicate that murine NK cells develop in the BM via our new four-stage model ex vivo and in vivo.

**Fig. 2:**
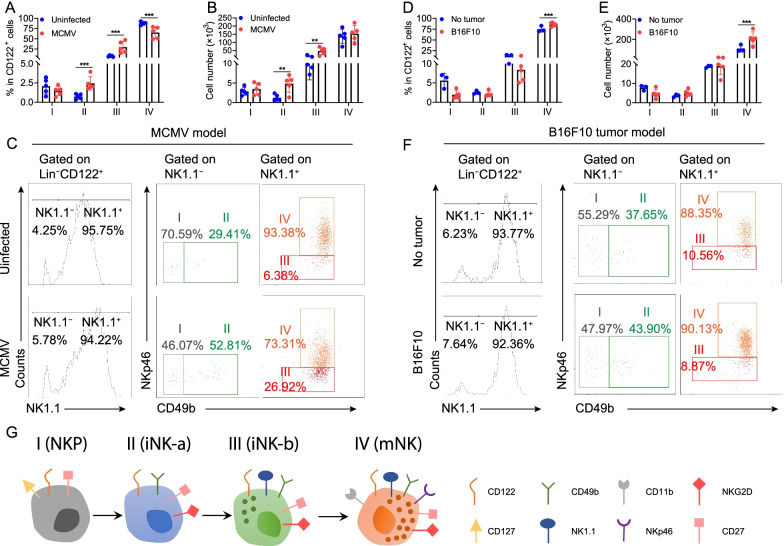
NK cell stage alternation in MCMV infection and B16F10 tumor models. For the viral infection model, C57BL/6 J mice were infected with an intraperitoneal injection of 2.5 × 10^4^ PFU MCMV. For the tumor-bearing model, B16F10 cells (1 × 10^5^) were injected intravenously into C57BL/6 J mice. **A**–**F** The percentages, absolute number, and representative dot plots of four stages of NK cells in BM from MCMV infection model (**A**–**C**, *n* = 5 per group) and B16F10 tumor model (**D**–**F**, *n* = 3–5 per group). **G** The new four-stage model for in vivo development of murine NK cells in the BM. Data are shown as mean ± SD and were analyzed by two-way ANOVA with Šídák post-test (**A**, **B**, **D**, **E**). Data are representative of at least three independent experiments. **P < 0.01; ***P < 0.001

We finally evaluated BM for the presence of the four NK cell populations associated with our new model during murine cytomegalovirus (MCMV) infection and tumor progression (Additional file 2). We found that MCMV infection mainly increased the percentage and absolute number of iNK-a/b cells (Fig. 2A–C), indicating that iNK cells are the main proliferation cells during MCMV infection. However, in B16F10 tumor-bearing mice, the percentage and absolute number of mNK cells were significantly upregulated in BM compared to those in mice without B16F10 (Fig. 2D–F), indicating that mNK cells increased in response to tumor triggers.

In summary, our study defines two iNK cell developmental stages and clarifies NKp46 as a mature NK cell marker in mouse BM. Our model includes the previously defined NK1.1^+^CD49b^−^ iNK cell population, and also the previously ignored NK1.1^−^CD49b^+^ population. Our findings fill an important gap in our understanding of the developmental steps linking NKP and mNK cells and answer a long-term question about where to place NK1.1^−^CD49b^+^ cells in the NK cell developmental map. Based on this, we propose a new four-stage model for in vivo development of murine NK cells in the BM (Fig. 2G). Although murine NK cell development mainly takes place in the BM, whether NK cell populations in other developmental sites also follow this model needs further investigation.

## Supplementary Information

**Additional file 1**. **Figure S1.** Functional characteristics of four developmental stages of murine NK cells.

**Additional file 2**. Materials and Methods. 

## Data Availability

All data generated during this study are included in this published article.

## Abbreviations

NK: Natural killer
BM: Bone marrow
CLPs: Common lymphoid progenitors
NKP: NK progenitor
iNK: Immature NK
mNK: Mature NK
MCMV: Murine cytomegalovirus
