# Supplementary material for: A four-stage model for murine natural killer cell development in vivo
Source: J Hematol Oncol. 2022 Mar 21;15:31. doi: 10.1186/s13045-022-01243-1 (PMC8935775; doi:10.1186/s13045-022-01243-1)
Supplement: Supplementary file 1 — Additional file 1. Figure S1. Functional characteristics of four developmental stages of murine NK cells. [file 13045_2022_1243_MOESM1_ESM.docx]

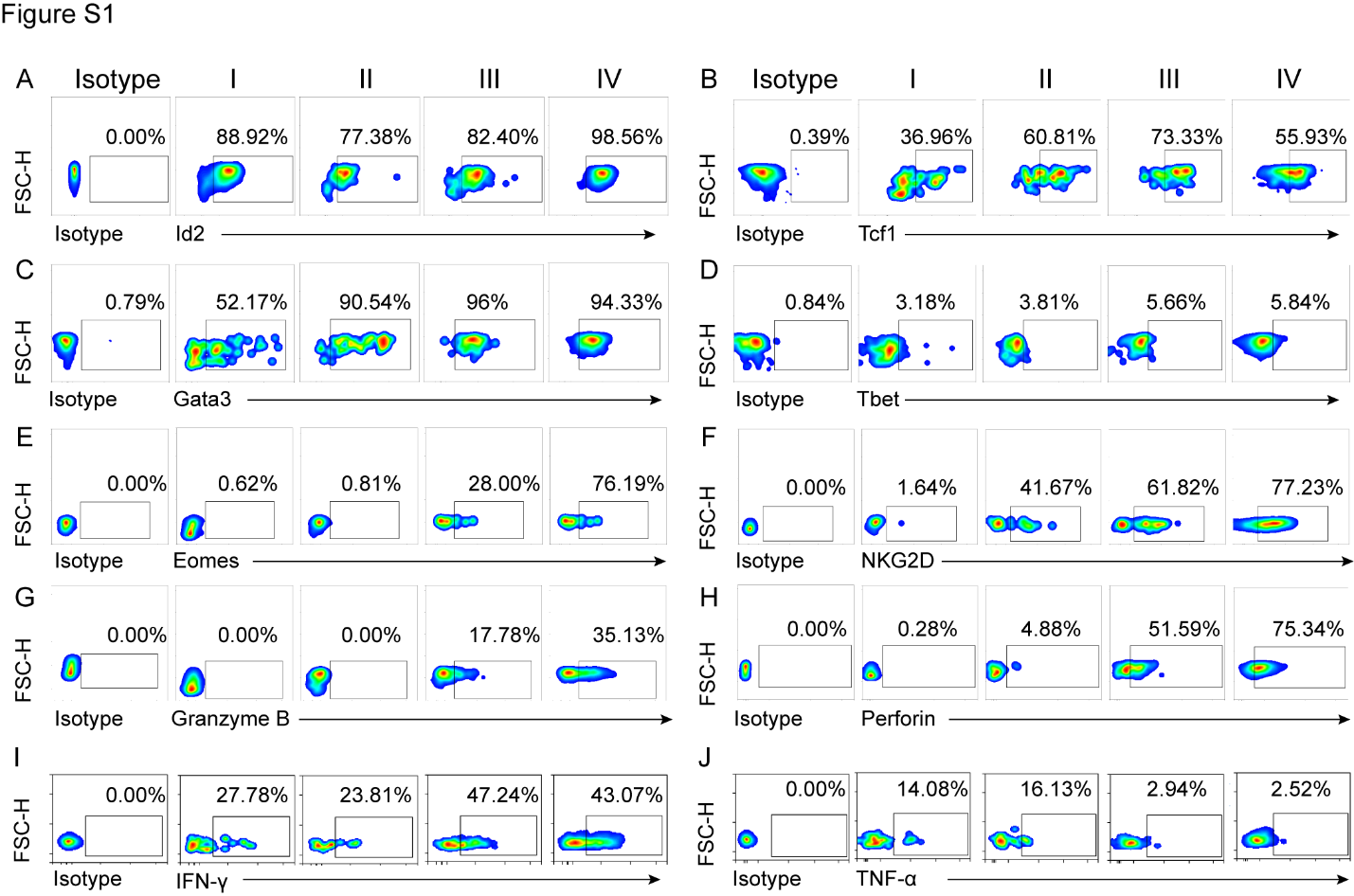


**Figure S1. Functional characteristics of four developmental stages of murine NK cells.** Single-cell suspension from bone marrow (BM) cells was prepared from wild-type mice and stained with indicated cell markers for flow cytometry. For examining IFN-γ and TNF-α, BM cells were stimulated with a leukocyte activation cocktail, with BD GolgiPlu for 4 h. Flow cytometry analysis of the transcription factors (A) Id2, (B) Tcf1, (C) Gata3, (D) Tbet, (E) Eomes, NK cell receptor (F) NKG2D, and NK cell effector molecules (G) granzyme B, (H) perforin, (I) IFN-γ, and (J) TNF-α in NK cell populations I-IV. Representative dot plots are shown. Summary data are presented in Figure 1E (n = 3).
