# Supplementary material for: A four-stage model for murine natural killer cell development in vivo
Source: J Hematol Oncol. 2022 Mar 21;15:31. doi: 10.1186/s13045-022-01243-1 (PMC8935775; doi:10.1186/s13045-022-01243-1)
Supplement: Supplementary file 2 — Additional file 2. Materials and Methods. [file 13045_2022_1243_MOESM2_ESM.docx]

**Material and Methods**

**Mice**

C57BL/6J mice were purchased from the Jackson Laboratory and housed at City of Hope. Congenic NCI B6-Ly5.1/Cr (CD45.1) mice were purchased from Charles River. *Rag2*^−/−^*Il2rg*^−/−^ were provided by the animal facility at City of Hope. NKp46^GFP/GFP^ mice and their heterozygous littermates (NKp46^+/GFP^) were used as previously described [1]. 6–12-wk-old male and female mice were used for the experiments. All experiments were approved by the City of Hope Institutional Animal Care and Use Committee.

**Flow cytometry and cell sorting**

BM cells were flushed from mice and a 70 μm strainer was used to generate a single-cell suspension. The cells were stained with antibodies for 30 min at 4°C in staining buffer (PBS containing 2% FBS) in the presence of a purified anti-mouse CD16/32 antibody (catalog #101302, Biolegend) to block non-specific binding. The cells were then washed twice and resuspended in staining buffer. The samples were analyzed using a Fortessa X20 flow cytometer (BD Bioscience) or sorted using an Aria Fusion cell sorter (BD Bioscience). Intracellular staining was performed by using CytoFix/CytoPerm buffer (BD Biosciences) or Foxp3/Transcription Factor Staining Buffer Set (eBioscience) according to the manufacturer’s instructions. Data were analyzed using NovoExpress software (ACEA Biosciences). The following fluorescence dye–labeled antibodies from BD Biosciences, BioLegend or Invitrogen were used: CD3ε (145-2C11), CD19 (1D3), Gr-1 (RB6-8C5), TER-119 (TER-119), CD11c (N418), CD122 (5H4), NK1.1 (PK136), CD49b (DX5), NKp46 (29A1.4), CD11b (M1/70), CD27 (LG.3A10), Id2 (ILCID2),Tcf1 (S33-966), Gata3 (L50-823), granzyme B (QA16A02), perforin (S16009A), NKG2D (CX5), Tbet (4b10), Eomes (WD1928), IFN-γ (XMG1.2), and TNF-α (MP6-XT22).

**Ex vivo differentiation assay**

NK cell populations I-IV from NKp46^+/GFP^ reporter mice were sorted and 5 × 10^4^ cells were seeded into a 96-well plate and cultured in the presence of IL-15 (50 ng/ml) for 14 days. The cells were then harvested and analyzed using flow cytometry.

**Adoptive transfer**

NK cell populations I-IV from CD45.1 mice were sorted and 1×10^4^ to 1×10^5^ cells were injected intravenously into *Rag2*^−/−^*Il2rg*^−/−^ mice. The presence of transferred cells was analyzed eight weeks after adoptive transfer.

**B16F10 melanoma model and murine cytomegalovirus (MCMV) infection model**

B16F10 cells (1 × 10^5^) were injected intravenously into C57BL/6J mice. Fourteen days after injection, the mice were euthanized for post-mortem analysis. The B16F10 cell line was provided by Dr. Hua Yu (City of Hope). For the viral infection model, C57BL/6J mice were infected with intraperitoneal injection of 2.5 × 10^4^ PFU MCMV (VR-1399; ATCC) as previously described [2]. Seven days after infection, the mice were euthanized for post-mortem analysis.

**Statistics**

The data are presented as mean ± SD and analyzed using two-way ANOVA for multiple comparisons. A p value less than 0.05 was considered statistically significant. The significance is indicated as *p<0.05; **p<0.01; ***p<0.001.

**SI References**

1. Wang, Y., et al., *Dependence of innate lymphoid cell 1 development on NKp46.* PLoS Biol, 2018. **16**(4): p. e2004867.

2. Ma, S., et al., *The RNA m6A reader YTHDF2 controls NK cell antitumor and antiviral immunity.* J Exp Med, 2021. **218**(8).
